# Effect of different NPK fertilization timing sequences management on soil-petiole system nutrient uptake and fertilizer utilization efficiency of drip irrigation cotton

**DOI:** 10.1038/s41598-023-40620-9

**Published:** 2023-08-31

**Authors:** Zhiqiang Dong, Yang Liu, Minghua Li, Baoxia Ci, Xi Lu, Xiaokang Feng, Shuai Wen, Fuyu Ma

**Affiliations:** 1 School of Agriculture, Shihezi University, Shihezi, 832003 Xinjiang People’s Republic of China; 2 Guiyang Healthcare Vocational University, Guiyang, 550081 Guizhou People’s Republic of China; 3 National and Local Joint Engineering Research Center of Information Management and Application Technology for Modern Agricultural Production (XPCC), Shihezi, 832003 Xinjiang People’s Republic of China

**Keywords:** Plant sciences, Plant ecology, Plant physiology

## Abstract

In order to elucidate the effects of different nitrogen (N), phosphorus (P), and potassium (K) fertilization timing sequences management on nutrient absorption and utilization in drip irrigation cotton, field experiments were conducted from 2020 to 2021. There are six timing sequences management methods for NPK fertilization (S1–S6: 1/3Time N–1/3Time PK–1/3Time W, 1/3Time PK–1/3Time N–1/3Time W, 1/2Time NPK–1/2Time W, 1/4Time W–1/4Time N–1/4Time PK–1/4Time W, 1/3Time W–1/3Time NPK–1/3Time W), among which S6 is the current management method for field fertilization timing sequences, and S7 is the non N. The results showed that during the main growth stage, S5 accumulated more nitrate nitrogen (NO_3_^–^-N) and ammonium nitrogen (NH_4_^+^-N) content in soil between 20 and 40 cm, and accumulated more available phosphorus content in soil between 5–15 cm and 15–25 cm, S5 reducing N leaching and increasing P mobility. It is recommended to change the timing sequences management method of NPK fertilization for drip irrigation cotton to 1/4Time W–1/4Time PK–1/4Time N–1/4Time W, which is beneficial for plant nutrient absorption and utilization while reducing environmental pollution.

Xinjiang is located in the arid zone of northwest China, where water resources and irrigation for farmland are scarce^1^. Along with water scarcity, agricultural production is harmed by indiscriminately applying large amounts of fertilizers^2^, resulting in low fertilizer efficiency and serious environmental pollution^3^. Water and fertilizer are considered the most important factors affecting cotton yield and quality^4^, therefore, research on cotton fertilization and irrigation research have received widespread attention worldwide^5,6^.

Nitrogen (N) and phosphorus (P) are important nutrients for crop growths^7,8^. Although N fertilizer is the most demanded in crop production, the environmental pollution problem caused by excessive application of N fertilizer is also becoming more serious^9^. Research have shown that the current farmland N fertilizer utilization rate is only about 30%, due to various reasons such as excessive application of N fertilizer, unreasonable irrigation management methods, crop types and soil properties, as well as the volatile and easy leaching characteristics of N fertilizer, the loss rate of N fertilizer is high. The rest of the nitrate nitrogen (NO_3_^–^-N) residue in the soil^10^, surface residue (0–40 cm) N can still be next crop reuse, but because the NO_3_^–^-N is not easy to soil colloidal adsorption^11^, residual part in rainfall or diffuse irrigation conditions of deep leaching loss, NO_3_^–^-N leaching in farmland soil is one of the important ways of N fertilizer loss and the main source of groundwater pollution, becoming an increasingly serious environmental pollution factor^12,13^. Unlike N, P is easily fixed and adsorbed in the soil, thus greatly reducing the efficiency of P use^14,15^. Therefore, reducing N leaching, increasing the displacement distance of P in soil, and exploring reasonable fertilization methods to promote the full use of N and P are urgent problems to be solved at present^16^.

Drip irrigation^17^ is a modern water-saving irrigation technology with controllable irrigation and fertilizer application in terms of time, sequence and quantity^18,19^. It can directly supply water and fertilizer to the soil near the crop roots at the right time and amount according to the crop's demand at different growth stages^20–22^, reduced losses such as leaching and volatilization of fertilizers, and significantly improving the efficiency of water and fertilizer utilization^22,23^. By applying water and fertilizer coupling, synchronization of the role of promoting fertilizer transformation, conducive to crop absorption and utilization^24,25^, fertilizer management is more efficient^26^. In the current cotton production in Xinjiang, the drip irrigation system in the field is usually about 1/3 time of water in one irrigation process, followed by N, P and potassium (K) fertilizer solution, and finally about 1/3 time of water in the drip to flush the pipe network, but the effect of this drip irrigation system is not ideal^26^.

To address this issue, this study utilizes perfect high-efficiency and water-saving drip irrigation technology in Xinjiang. Using the advantages of drip irrigation in terms of controllable fertilization and irrigation timing sequences, six different fertilization timing sequences management of NPK were established. Explore the impact of different fertilization timing sequences management on nutrient absorption and fertilizer utilization efficiency in the soil petiole system, and find the optimal NPK fertilization timing sequences management method.

## Materials and methods

### Materials

The experiment was conducted in 2020–2021 at the teaching trial site of Shihezi University, Shihezi City, Xinjiang Uygur Autonomous Region (44°18′N, 86°02′E). The cotton variety used in this study was Lumianyan 24, selected by Shandong Cotton Research Center, which is a medium-late maturing variety with a fertility period of about 130 days. Fertilization and weeding of plants in the present study complies with international, national and institutional guidelines.

### Experimental design

The experiment was designed with six treatments (Table 1) with three replications randomized in completely zoned groups, planting pattern: 1 film, 3 rows and 3 strips, mulching, each plot 20 m long, row spacing 0.66 m. In all treatments (Table 1), S6 is the conventional drip irrigation (The drip irrigation is surface) fertilization timing sequences management and S7 is the treatment without N fertilization, where W denotes water drip, N denotes N drip, and PK denotes P and K drip. In which 1/3 time N–1/3 time PK–1/3 time W (S1), means the whole drip irrigation fertilization process, 1/3 of the total time of drip irrigation dripping N fertilizer solution first, followed by 1/3 of the total time of drip irrigation dripping P and K fertilizer solution, 1/3 of the total time of drip irrigation dripping water last, and the rest is the same. The application rates of N, P and K were 300 kg/ha, 109.8 kg/ha (P_2_O_5_) and 91.8 kg/ha (K_2_O), respectively. The types of fertilizers used as sources of N, P and K were urea, KH_2_PO4 and K_2_SO_4_, all of them are special fertilizer for drip irrigation with excellent water solubility. Eight (2020) and nine (2021) times during the cotton growth stages, with the ratio of 2:8 for N fertilizer and 3:7 for P and K fertilizer between bud stage (BS) and flowering and bolling stage (FABS), and the total amount of N, P and K fertilizer applied in a single application was the same for each plot (Table 2). The total amount of N, P and K fertilizer was the same for each plot.

**Table 1:** NPK fertilization timing sequences.

Processing number	Fertilization timing sequences
S1	1/3Time N–1/3Time PK–1/3Time W
S2	1/3Time PK–1/3Time N–1/3Time W
S3	1/2Time NPK–1/2Time W
S4	1/4Time W–1/4Time N–1/4Time PK–1/4Time W
S5	1/4Time W–1/4Time PK–1/4Time N–1/4Time W
S6 (CK)	1/3Time W–1/3Time NPK–1/3Time W
S7	1/3Time W–1/3Time PK–1/3Time W

**Table 2:** Fertilization proportion.

Date		Order	Fertilization%		
			N%	P_2_O_5_%	K_2_O%
2020	4–22	1	–	–	–
	6–08	2	10.0	15.0	15.0
	6–17	3	10.0	15.0	15.0
	6–27	4	13.3	11.7	11.7
	7–08	5	13.3	11.7	11.7
	7–18	6	13.3	11.7	11.7
	7–24	7	13.3	11.7	11.7
	8–02	8	13.3	11.7	11.7
	8–12	9	13.3	11.7	11.7
	8–20	10	–	–	–
	Total		100	100	100
2021	4–27	1	–	–	–
	6–11	2	6.7	10.0	10.0
	6–20	3	6.7	10.0	10.0
	6–27	4	6.7	10.0	10.0
	7–04	5	13.3	11.7	11.7
	7–11	6	13.3	11.7	11.7
	7–18	7	13.3	11.7	11.7
	7–28	8	13.3	11.7	11.7
	8–08	9	13.3	11.7	11.7
	8–15	10	13.3	11.7	11.7
	8–22	11	–	–	–
	Total		100	100	100

### Determination of NO_3_^–^N, NH_4_^+^-N and AP content in soils

Soil samples were collected under the drip irrigation belt and between the planting rows at the full bud stage (FBS), full flowering stage (FFS), peak boll stage (PBS) and boll opening stage (BOS) of cotton, respectively, and soil NO_3_^–^-N and ammonium nitrogen (NH_4_^+^-N) were measured at depths of 0–20 cm, 20–40 cm and 40–60 cm. Soil available phosphorus (AP) was measured at sampling depths of 0–5 cm, 5–15 cm, and 15–25 cm.

Determination of NO_3_^–^-N and NH_4_^+^-N in soil: The samples were collected and stored immediately on ice. Before measurement, thaw the sample. Immediately after thawing, thoroughly mix the sample through a 2 mm sieve, weigh 5 g of soil sample, add 50 mL of 2 mol/L KCl solution, shake for 30 min, and filter. The extract is immediately frozen and stored. The extracts were thawed before measurement, and the NO_3_^–^-N and NH_4_^+^-N contents of the soils were determined using a flow analyzer.

### Determination of NO_3_^–^-N and PO_4_^3–^-P content in petioles

Since a large number of petioles need to be removed to test the nutrient content of cotton petioles, the plot area was divided into two parts in the ratio of 3:2, where 3/5 plot area (SCA1) was used for plant sampling, and 2/5 plot area (SCA2) was used for petiole sampling.

Sampling is generally carried out on sunny days from 12:00 to 14:00. Cotton metabolism is in dynamic equilibrium during this period, and the stored NO_3_^–^N, inorganic phosphorus (PO_4_^3–^-P) content in the body best reflects the relative relationship between nutrient uptake and assimilation. The content of NO_3_^–^N, PO_4_^3–^-P in cotton petiole is relatively stable, which can reflect the authenticity of nutrient content in cotton petiole during the important reproductive period of cotton.

Ten petioles with leaves were collected at fixed points in each SCA2 plot, and their leaves were removed leaving only the petioles. The samples were washed with distilled water, and the petioles and leaves were separated. The petioles were cut and pressed. The contents of NO_3_^–^-N in cotton petioles were determined using LAQUA Twin NO_3_^-^ meter and K^+^ meter (HORIBA Inc., Japan), while the PO_4_^3–^-P content was determined using RQflex20 Reflectoquant (Merck Inc., Germany).

### Plant N content and yield determination

Three uniformly growing cotton plants were taken from each SCA1 plot and broken down into three parts: leaves, stems and reproductive organs from above the cotyledons. The dried cotton stems, leaves and reproductive organs were crushed and passed through 0.5 mm sieve. The N content of each organ was determined by the Kjeldahl method using sulfuric acid digestion.

The number of harvested plants and the number of spatted bolls in an area of 3 m × 2.28 m were counted in each plot selected for 3 m length during the harvesting period, after which 50 spatted bolls were harvested in each plot, weighed and the single boll weight was calculated. The seed cotton yield was obtained according to the yield calculation formula.

### Data processing

Significance of elements under different NPK fertilization timing sequences management was evaluated by using one-way analysis of variance (ANOVA) and Duncan test. Significance was reported at *P* < 0.05 level. All data were analyzed with SPSS 27.0 statistical software (SPSS, Inc., Chicago, IL, USA) and the graphs were drawn by using Origin 2018 (Origin Lab Corporation, Northampton USA).

N recovery efficiency^27^ (NRE%) = (N uptake in N application area − N uptake in no N application area)/N application.

N fertilizer agronomic utilization efficiency^28^ (aNUE kg kg^−1^) = (Yield in N application area − Yield in no N application area)/N application.

Fertilizer Partial productivity^28^ (FPP kg kg^−1^) = Yield/Fertilizer input.

## Results

### Effect of different NPK fertilization timing management on N uptake in soil-petiole system

The effect of different NPK fertilization timing sequences management on the NO_3_^–^-N content of different soil layers under cotton is shown in Table 4. With the change of the cotton growth period, the soil NO_3_^–^-N content of each treatment decreased rapidly at the FBS, increased again at the FFS, was relatively low at the PBS, and increased slightly at the BOS. Soil NO_3_^–^-N content was more depleted during the FBS and PBS, and the overall soil NO_3_^–^-N content in 2021 was slightly higher than that in 2020.

Under different NPK timing sequences management, the S1 and S3 showed a trend of increasing soil NO_3_^–^-N content with increasing the same soil depth. S4 showed the same trend as S1 and S3 at FBS (2021), FFS and PBS, while S2, S5 and S6 showed a higher concentration of soil NO_3_^–^-N in the 0–20 cm layer. S5 accumulates more in the 20–40 cm soil layer during all important growth stages.

The significance of soil NO_3_^–^-N content in different soil layers under different NPK fertilization timing sequences management was analyzed (Table 3). In the 20–40 cm soil layer, the overall performance of soil NO_3_^–^-N content is S5 > S2 > S6 > S4 > S3 > S1 > S7. The soil NO_3_^–^-N content of S5 is significantly higher than that of other treatments and significantly higher than that of other treatments. At the FFS, the soil NO_3_^–^-N content of S5 is 9.4% (2021) higher than that of S6 and 23.7% (2021) higher than that of S7. In 40-60 cm soil layer, the overall content of soil NO_3_^–^-N is S3 > S1 > S4 > S6 > S2 > S5 > S7. The content of soil NO_3_^–^-N in S3 and S1 is significantly higher than that in other treatments. In individual growth stages, the content of soil NO_3_^–^-N in S4 is also significantly higher than that in other treatments, except that it has no significant difference with S1 and S3. N application was significantly higher than no N application. The content of soil NO_3_^–^-N in each treatment had strong significance in FBS (except 2020), FFS and PBS, and decreased significantly among treatments at BOS.

**Table 3:** Significance of NO_3_^–^-N content in different soil layers under different fertilization timing sequences in 2020–2021.

Growth stage	Soil depth	2020 Soil NO_3_^–^-N content (mg/kg)							2021 Soil NO_3_^–^-N content (mg/kg)						
		S1	S2	S3	S4	S5	S6	S7	S1	S2	S3	S4	S5	S6	S7
FBS	0–20	16.44a	16.15a	17.17a	16.25a	15.85a	15.92a	14.47a	16.81d	18.31b	17.75c	18.11bc	19.10a	19.16a	15.93e
	20–40	18.87a	19.15a	19.24a	19.40a	18.21a	18.58a	16.74a	20.29b	18.12d	18.40d	19.11c	21.31a	19.30c	17.25e
	40–60	17.85a	16.42a	17.76a	16.53a	16.45a	16.34a	16.60a	20.33b	18.94d	21.49a	19.76c	17.37e	19.06d	16.14f
FFS	0–20	18.78de	19.53ab	18.49e	19.05cd	19.21bc	19.81a	17.37f	18.75bc	20.07a	19.26b	19.26b	20.50a	20.70a	17.55d
	20–40	19.95d	21.28b	20.25d	20.85c	22.30a	21.24b	19.10e	20.43e	22.64b	20.77de	21.33cd	24.14a	22.07bc	19.51f
	40–60	21.54b	19.01d	22.18a	20.31c	18.26e	19.22d	17.19f	22.72b	19.99d	23.43a	21.05c	19.20e	20.12d	18.04f
PBS	0–20	15.22d	16.20bc	15.13d	15.94c	16.58ab	16.71a	14.25e	15.84d	18.26a	15.28e	16.28cd	17.34b	18.64a	14.27f
	20–40	16.84e	18.32ab	17.14de	17.66cd	18.81a	18.14bc	16.06f	17.13d	19.24b	17.28d	18.36c	20.66a	18.96b	16.48e
	40–60	18.15a	16.89b	18.31a	17.92a	16.23c	17.24b	15.12d	20.33b	16.90d	21.34a	18.38c	16.37e	17.29d	15.16f
BOS	0–20	17.26b	17.68ab	17.15b	17.55ab	16.96b	18.00a	17.05b	17.98ab	18.94ab	18.31ab	18.75ab	18.92ab	19.04a	16.82b
	20–40	18.58bc	19.67a	18.71b	19.20a	19.53a	19.33a	18.23c	20.77a	20.45a	20.98a	21.31a	21.42a	21.06a	18.44b
	40–60	18.12b	17.62cd	18.83a	18.35b	17.51d	17.98bc	17.45d	20.41a	17.92b	21.02a	17.48b	17.22b	17.73b	16.75b

Different lowercase letters mean significant differences (*P* < 0.05) among the different fertilization timing sequence under the same soil layer.

The soil NH_4_^+^-N content showed a general trend of gradually decreasing with increasing soil depth under different NPK fertilization timing sequences management, but the soil NH_4_^+^-N content was significantly lower than the NO_3_^–^-N content.

The trend of soil NH_4_^+^-N content in each treatment under different NPK fertilization timing sequences management is shown in Table 4. S1 showed a trend of decreasing and then increasing soil NH_4_^+^-N content with increasing soil depth at the FFS and PBS, where the soil NH_4_^+^-N content peaked at 40–60 cm soil layer at the PBS, showing a trend of gradually deepening soil NH_4_^+^-N content with deepening soil layer. The S4 also showed a decreasing trend followed by an increasing trend in the FBS (2021), PBS (2021) and BOS (2021) stages. The NH_4_^+^-N content of soil in S2 was higher than that of other treatments except for S5, which showed a trend of increasing and then decreasing with the increase of soil depth in S5 at FFS and PBS stage (2021), and reached the peak in 20–40 cm soil layer.

**Table 4:** Significance of NH_4_^+^-N content in different soil layers under different fertilization timing sequences in 2020–2021.

Growth stage	Soil depth	2020 Soil NH_4_^+^-N content (mg/kg)							2021 Soil NH_4_^+^-N content (mg/kg)						
		S1	S2	S3	S4	S5	S6	S7	S1	S2	S3	S4	S5	S6	S7
FBS	0–20	6.32b	6.74a	6.41b	6.24b	6.34b	6.79a	4.22c	7.09e	8.87a	6.85e	7.35d	7.90c	8.43b	6.33f
	20–40	5.77a	5.68ab	5.66abc	5.27d	5.42bcd	5.39cd	4.07e	6.83b	6.33cd	6.77b	6.58bc	7.51a	7.33a	6.08d
	40–60	5.40ab	4.47d	4.98c	5.66a	4.29de	5.22bc	3.97e	6.80a	6.18b	6.67a	6.38ab	6.41ab	6.11b	5.45c
FFS	0–20	7.32d	8.19b	7.15d	7.81c	8.02bc	8.72a	6.31e	9.62c	10.81a	9.38c	10.04b	10.11b	10.29b	8.39d
	20–40	6.30d	7.77b	7.75b	7.13c	8.27a	6.85c	5.97d	8.07d	9.17b	7.35e	8.29d	9.56a	8.82c	7.19e
	40–60	7.39b	5.30d	7.83a	6.24c	6.02c	5.96c	5.05d	8.79ab	8.18c	8.95a	8.46bc	7.52d	8.25c	7.27d
PBS	0–20	4.55d	5.38b	4.73cd	4.93c	5.32b	6.25a	4.27e	6.95de	7.60b	6.77e	7.16cd	7.39bc	8.67a	6.24f
	20–40	4.13b	4.32b	4.95a	4.87a	5.03a	3.34c	3.06c	6.43c	6.67c	6.28c	7.06b	7.88a	6.56c	5.57d
	40–60	5.21a	4.21b	5.23a	4.35b	4.16b	3.35c	2.38d	7.74a	6.83c	7.95a	7.28b	6.25d	7.05bc	5.73e
BOS	0–20	8.04a	8.09a	7.95a	8.06a	8.07a	8.08a	7.82a	9.17bc	9.60ab	9.18bc	9.28ab	9.31ab	9.63a	8.84c
	20–40	6.73c	6.92bc	7.16b	6.87bc	7.65a	6.38d	6.38d	8.12b	8.38b	7.61c	8.09b	9.23a	8.16b	7.61c
	40–60	5.90a	5.93a	6.06a	5.99a	5.94a	5.78a	4.86b	8.64a	8.15b	8.69a	8.17b	7.97b	8.13b	7.08c

Different lowercase letters mean significant differences (*P* < 0.05) among the different fertilization timing sequence under the same soil layer. FBS, FFS, PBS and BOS represent full bud stage, full flower stage, peak boll stage and boll opening stage, respectively.

The significance of soil NH_4_^+^-N content in different soil layers under different NPK fertilization timing sequences management was analyzed (Table 4). In the 0**–**20 cm soil layer, the soil NH_4_^+^-N content of the S6 was significantly higher than the other treatments and not significantly different from the S2 and S5. In the 20**–**40 cm soil layer, the soil NH_4_^+^-N content of S5 was significant with other treatments, and the S5 increased 6.4%-38.5% (2020) and 4.3%-33.0% (2021) compared with other treatments at the FFS, and 20.7% (2020) and 8.4% (2021) compared with S6 (CK), and S5 increased S2 had significantly higher soil NH_4_^+^-N content at FFS, PBS and BOS stages than the other treatments, except for the S5, which was lower than the S5. In the 40**–**60 cm soil layer, the soil NH_4_^+^-N content of S3 and S1 was significantly higher than the other treatments, and S4 was also significantly higher than the other treatments in individual growth stages, except that it was not significant with S1 and S3. The soil NH_4_^+^-N content was significantly higher in the N application than in the non-N application. The soil NH_4_^+^-N content of each treatment was highly significant at the FBS, FFS and PBS, and the difference between treatments decreased when the BOS was reached.

**Figure 1:**
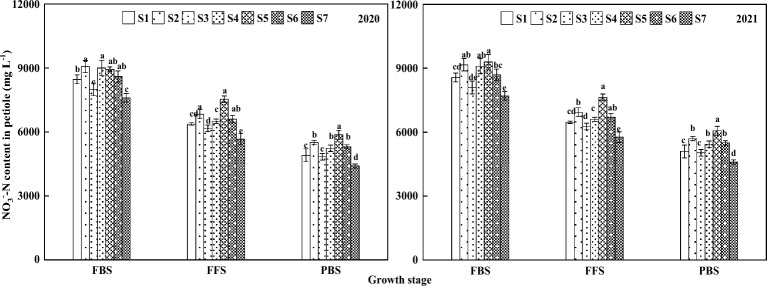
Changes of NO_3_^–^-N content in petioles under different NPK fertilization timing sequences management in important growth stages in 2020–2021. Different letters indicated significant difference at 5% probability level in the same observation period.

The petiole NO_3_^–^-N content of functional leaves of cotton showed a trend of gradual decrease with the advancement of the growth stages, reaching a maximum at the FBS and a minimum at the PBS (Fig. 1). 2020, under the management of different NPK fertilization timing sequences management, the petiole NO_3_^–^-N content at the FBS was S4 > S2 > S5 > S6 > S1 > S3 > S7, among which the difference between the petiole NO_3_^–^-N content of S2, S4, S5 and S6 was not significant. S2 and S4 were significantly higher than S1, S3 and S7, no difference between S1, S5 and S6, and significantly higher than S1, S3 and S7. The FFS showed S5 > S2 > S6 > S4 > S1 > S3 > S7, no significant difference between S2, S5 and S6 in petiole NO_3_^–^-N content, but all were significantly higher than other treatments, no significant difference between S1 and S4, difference between S1 and S3 was not significant, and all treatments were significantly higher than S7. At the PBS it showed S5 > S2 > S6 > S4 > S1 > S3 > S7. The NO_3_^–^-N content of petiole was significantly higher in S5 than in other treatments, the difference between S2, S4 and S6 was not significant, and the difference between S1 and S3 was not significant, but both were significantly higher than in S7.

Under different NPK fertilization timing sequences management in 2021, the petiole NO_3_^–^-N content at the bloom stage showed S5 > S2 > S4 > S6 > S1 > S3 > S7, among which the difference in petiole NO_3_^–^-N content among S2, S4 and S5 was not significant, and S5 was significantly higher than S1, S3, S6 and S7. The significant performance at the FFS and PBS stage was consistent with the trend in 2020.

### Effect of different NPK fertilization timing management on P uptake in soil-petiole system

With the change of growth stage, the soil AP content was the highest at the FBS, decreased rapidly at the FFS, was the lowest at the PBS and increased at the BOS under different NPK fertilization timing sequences management. The soil AP content decreased gradually with the increase of soil depth from the FBS to the BOS. As with soil NO_3_^–^-N and NH_4_^+^-N, the overall soil AP content was higher in 2021 than in 2020.

The trend of soil AP content of each treatment under different NPK fertilization timing sequences management is shown in Table 5. S1, S3, S4 and S6 have more soil AP content aggregated in 0**–**5 cm. S5 has the highest content in 5**–**15 cm and 15**–**25 cm soil layer. The S5 had higher soil AP content at FFS (2020) and 15**–**25 cm soil layer at PBS than at 5**–**15 cm soil layer, while the S7 had lower soil AP content than the other treatments.

**Table 5:** Significance of AP content in different soil layers under different fertilization timing sequences in 2020–2021.

Growth stage	Soil depth	2020 Soil AP content (mg/kg)							2021 Soil AP content (mg/kg)						
		S1	S2	S3	S4	S5	S6	S7	S1	S2	S3	S4	S5	S6	S7
FBS	0–5	24.89bc	26.54a	26.84a	25.37b	24.36b	24.94bc	25.49b	28.81ab	27.11de	27.59cd	29.06a	26.91e	28.23bc	27.91c
	5–15	22.33b	21.49cd	21.04d	21.89bc	22.88a	22.30b	21.14d	24.06bc	24.31b	23.88bcd	23.57d	25.11a	24.13bc	23.69cd
	15–25	19.58b	18.74de	18.59e	19.10cd	20.57a	19.36bc	18.38e	20.85c	21.97b	21.59b	20.70c	22.75a	21.85b	20.92c
FFS	0–5	22.68ab	21.28de	21.58cde	22.88a	21.05e	22.14bc	21.81cd	24.77ab	24.14c	24.06c	25.11a	23.21d	23.75cd	24.26bc
	5–15	17.95d	19.28b	18.25d	18.85c	20.34a	19.24b	17.10e	20.71cd	22.03ab	21.21c	20.63d	22.42a	21.79b	20.27d
	15–25	17.42cd	18.49b	17.67c	16.75e	20.64a	18.03bc	16.99de	18.53cd	19.32b	18.82bc	18.17d	20.78a	19.23b	17.99d
PBS	0–5	20.69a	19.66c	20.50ab	20.82a	19.00d	19.24d	20.18b	22.91a	21.46d	22.72ab	23.04a	21.22d	21.88c	22.40b
	5–15	17.78cd	18.32ab	17.14e	16.84e	18.74a	18.14bc	17.35de	18.58cd	20.21a	19.22bc	18.36d	20.03a	19.51ab	18.06d
	15–25	16.67de	18.09b	17.14cd	16.37e	18.94a	17.53c	15.67f	17.56d	19.16b	18.42c	17.26d	20.58a	18.83bc	16.56e
BOS	0–5	22.90ab	22.49b	22.37b	23.23a	22.19b	22.78ab	22.27b	25.77ab	25.44ab	25.57ab	25.89a	24.89c	25.72ab	25.38b
	5–15	20.87a	21.33a	20.38b	20.24bc	21.20a	20.99a	19.90c	23.09a	22.47bc	23.22a	22.60b	23.55a	23.42a	22.12c
	15–25	20.06c	20.72b	20.42b	19.96c	21.52a	20.57b	19.86c	22.40de	23.16b	22.76cd	22.29e	23.87a	22.90bc	22.19e
AVE	0–5	22.79	22.49	22.82	23.08	21.65	22.28	22.44	25.57	24.54	24.99	25.78	24.058	24.90	24.99
	5–15	19.73	20.11	19.20	19.46	20.79	20.17	18.87	21.61	22.26	21.88	21.29	22.78	22.21	21.04
	15–25	18.43	19.01	18.46	18.05	20.42	18.87	17.73	19.84	20.90	20.40	19.61	22.00	20.70	19.42

Different lowercase letters mean significant differences (*P* < 0.05) among the different fertilization timing sequence under the same soil layer. FBS, FFS, PBS and BOS represent full bud stage, full flower stage, peak boll stage and boll opening stage, respectively. AP, AVE represent soil available phosphorus and the average value of soil AP content.

The significance of soil AP content in different soil layers under different NPK fertilization timing sequences management was analyzed (Table 5). In the 0**–**5 cm soil layer, the S4 was significantly higher than the other treatments at the FFS, PBS and BOS. In the 5**–**15 cm soil layer, the soil AP content of S5 was significant and highest compared with other treatments, and the average soil AP content of S5 increased by 3.1% (2020) and 2.6% (2021) compared with S6 (CK). In the 15**–**25 cm soil layer, the soil AP content of S5 was also significantly higher than the other treatments, and the soil AP content of S5 increased 11.6%**–**23.2% (2020) and 7.6%**–**15.5% (2021) compared with the other treatments at the FFS, and 20.7% (2020) and 8.4% (2021) compared with S6 (CK) at the bell bloom stage soil AP content increased by 4.7%**–**20.9% (2020), 7.4%**–**24.3% (2021) compared to other treatments and 8.0% (2020), 9.3% (2021) compared to S6 (CK).The average soil AP content increased by 8.2% (2020), 6.3% (2021) in S5 compared to S6 (CK). S5 significantly enhanced the mobility of soil phosphorus to deeper soil layers.

**Figure 2:**
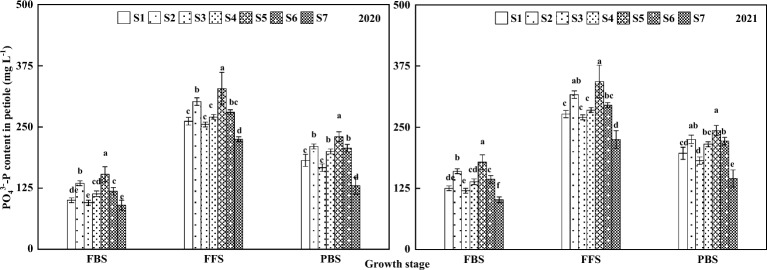
Changes of PO_4_^3–^-P content in petioles under different NPK fertilization timing sequences management in important growth stages in 2020–2021. Different letters indicated significant difference at 5% probability level in the same observation period.

The trend of PO_4_^3–^-P content of cotton petioles under different NPK fertilization timing sequences management is shown in Fig. 2, which shows a trend of increasing and then decreasing as the growth stage progresses, reaching a maximum at the FFS and a minimum at the FBS. In 2020, at FBS, petiole PO_4_^3–^-P content was significantly higher in S5 than in other treatments, and S2 was also significantly higher than in other treatments. The difference between S4 and S6 was not significant, and the difference between S1 and S4 was not significant. S6 was significantly higher than S1, S3 and S7, and there was no significant difference between S1, S3 and S7. At FFS, petiole PO_4_^3–^-P content was significantly higher in S5 than in other treatments, and S2 was significantly higher than in other treatments, except no significant difference between S1, S3 and S4, and all treatments were significantly higher than in S7. At PBS, petiole PO_4_^3–^-P content was significantly higher in the S5 than in the other treatments, S2 was significantly higher than in the other treatments, except for no significant difference with the S6, all were significantly higher than in the other treatments, S1, S3 and S4 were not significantly different from each other, and all were significantly higher than in S7. All treatments were significantly higher than the S7.

In 2021, at FBS, the trend of PO_4_^3–^-P content of petiole was the same as in 2020, except that S1 and S3 had significantly higher PO_4_^3–^-P content than S7. At FFS, S5 had significantly higher PO_4_^3–^-P content of petiole than other treatments, except for no significant difference with S2. S6 were not significantly different from each other, and all treatments were significantly higher than S7. At the PBS, the PO_4_^3–^-P content of petiole of S5 was significantly higher than other treatments except for no significant difference with S2. There was no significant difference between S2, S4 and S6, but S2 and S6 were significantly higher than S1, S3 and S7. The difference between S1 and S4 was not significant, and the difference between S1 and S3 was not significant. All treatments were significantly higher than S7.

### Effect of different NPK fertilizer fertilization timing sequences management on fertilizer utilization efficiency

Different NPK fertilizer fertilization timing sequences management significantly affected fertilizer utilization efficiency (Table 6). 2020, N fertilizer recovery efficiency (NRE) under different NPK fertilizer fertilization timing sequences management showed S5 > S2 > S6 > S4 > S1 > S3, where NRE under S5 was significantly highest among the other treatments, the difference between S2, S4 and S6 was not significant, S2 and S6 were significantly higher than S1 and S3. The difference between S4 and S1 was not significant but significantly higher than S3, and the difference between S1 and S3 was not significant. N fertilizer agronomic utilization efficiency (aNUE) was significantly higher in S5 followed by S2 which was also significantly higher than other treatments. The difference between S4 and S6 was not significant and S6 was significantly higher than S1 and S3. In terms of fertilizer partial productivity (FPP) significance, S5 was significantly higher than the other treatments, and S2 was also significantly higher than the other treatments, except for no significant difference with S5. NRE was 58.5% and 24.0% higher and aNUE was 55.9 and 76.6% higher under S5 compared to S6 (CK).

**Table 6:** Fertilizer utilization efficiency as influenced by different NPK fertilization timing sequences management in 2020–2021.

Year	Treatment number	NRE%	aNUE (kg kg^−1^)	PPF (kg kg^−1^)
2020	S1	17.0cd	1.64de	10.96e
	S2	28.8b	3.12b	11.85b
	S3	12.3d	1.40e	10.82e
	S4	21.1bc	2.03cd	11.19d
	S5	40.9a	3.71a	12.20a
	S6	25.8b	2.38c	11.40c
	S7	–	–	9.98f
2021	S1	23.1bc	1.80c	10.89d
	S2	34.6a	3.40b	12.15b
	S3	15.1c	1.52c	10.76d
	S4	28.0b	2.05c	11.14d
	S5	42.9a	4.45a	12.77a
	S6	27.0bc	2.52bc	11.62c
	S7	–	–	10.11e

Different letters indicated significant difference at 5% probability level.

## Discussion

The distribution law of soil NO_3_^–^-N and NH_4_^+^-N is affected by numerous factors. Different fertilization timing sequences of N, P, and K are critical in NO_3_^–^-N and NH_4_^+^-N distribution, and the contents of soil NO_3_^–^-N and NH_4_^+^-N directly affect absorption and utilization of plant nutrients^29^. Due to the characteristics of drip irrigation and fertilization with water, nutrients are also concentrated in the moist body formed by dripping water. According to research N fertilizer exhibits high solubility and good mobility in soil^30^. This study confirmed that when N fertilizer was applied in the early stage of drip irrigation process, soil NO_3_^–^-N and NH_4_^+^-N contents significantly increased in 40–60 cm soil layer. Among them, S1 and S3 treatments demonstrated a trend of increasing soil NO_3_^–^-N and NH_4_^+^-N content with increasing soil depth, increasing the risk of NO_3_^–^-N and NH_4_^+^-N leaching^31^. When N fertilizer is applied in the middle and early stages of drip irrigation, the contents of soil NO_3_^–^-N and NH_4_^+^-N contents significantly increase in 0–20 cm soil layer, and S2 and S6 concentrations in the upper soil are the most obvious, which is not conducive to better absorption of nutrients by cotton roots. When N fertilizer is applied in the middle and late stages of drip irrigation, soil NO_3_^–^-N and NH_4_^+^-N contents significantly increase in 20–40 cm. Among them, soil NO_3_^–^-N and NH_4_^+^-N contents in S5 are significantly higher than in other treatments, indicating that appropriate backward fertilization timing sequences of N fertilizer can adjust the distribution of soil NO_3_^–^-N and NH_4_^+^-N and make it aggregate in the middle soil layer, which helps cotton roots better absorb and utilize N and improve N utilization efficiency. The indoor simulation results of Li et al^32,33^, indicated that medium-term fertilization treatment (1/4 Time W–1/2 Time N–1/4 Time W) improved N distribution uniformity in soil. Gardenas et al^34^, selected four micro-irrigation and fertilization systems in 4 different soil types to form 16 cases, and simulated the effect of system operation mode on NO_3_^–^-N leaching. The simulation results reveal that fertilization in the later stage of irrigation process can reduce the leaching loss of NO_3_^–^-N yielding, similar conclusions during this test.

P is mainly adsorbed by the soil, with poor mobility. When P comes in contact with the soil, adsorption, fixation, and chemical reaction fixation occur^35^, resulting in P is fixation as ineffective state P fertilizer for plant uptake and utilization^36^. As a result, P is subjected to stronger adsorption by the soil, affecting the amount and the rate of migration^37^. According to changes in AP content in different soil depths under various NPK timing sequences, it can be seen that during drip irrigation process, in the process of drip irrigation, with the postponement of the fertilization timing sequence of P fertilizer, the soil AP accumulates more at 0–5 cm, among which soil AP content under S4 was most concentrated in 0–5 cm soil layer, which was significantly higher than other treatments. Soil AP content was the lowest in 5–15 cm and 15–25 cm soil layers. This indicates that directly application P into the soil during drip irrigation does not promote downward migration of soil AP content. In contrast, in the drip irrigation process, the soil AP content was more concentrated in 5–15 cm and 15–25 cm soil layers when water was applied for a period of time before applying P, at the same time, the plant can absorb P better, and the PO_4_^3–^-P content of petiole is also higher. In particular, soil AP content of S5 was significantly higher than other treatments. This implies that according to the law of P fertilizer transport in soil, irrigating water for a period of time before applying P during in the drip irrigation process improved P mobility in the soil^38^. This may be because the drip irrigation process with water first activates the organic matter and microorganisms in the soil, reducing P fixation by the soil and activating insoluble P compounds^39^, due to changing the timing sequences of drip irrigation fertilization, placing P fertilizer earlier in the soil is beneficial for the movement of P fertilizer towards a deeper layer based on the characteristics of the fertilizer following the water during the drip irrigation process.

## Conclusion

In summary, this work confirms that the drip irrigation cotton NPK fertilization timing sequences management method of S5 (1/4Time W-1/4Time PK-1/4Time N-1/4Time W), which prevents N leaching from the cotton soil, increases P displacement distance, benefits plant nutrient absorption, significantly improves fertilizer utilization efficiency, and reduces environmental pollution.

## Data Availability

The datasets used or analysed during the current study are available from the corresponding author on reason able request.
